# Selective Limbic Blood–Brain Barrier Breakdown in a Feline Model of Limbic Encephalitis with LGI1 Antibodies

**DOI:** 10.3389/fimmu.2017.01364

**Published:** 2017-10-18

**Authors:** Anna R. Tröscher, Andrea Klang, Maria French, Lucía Quemada-Garrido, Sibylle Maria Kneissl, Christian G. Bien, Ákos Pákozdy, Jan Bauer

**Affiliations:** ^1^Department of Neuroimmunology, Center for Brain Research, Medical University of Vienna, Vienna, Austria; ^2^Department for Pathobiology, Institute of Pathology and Forensic Veterinary Medicine, University of Veterinary Medicine, Vienna, Austria; ^3^Diagnostic Imaging, Department for Companion Animals and Horses, University of Veterinary Medicine, Vienna, Austria; ^4^Epilepsy Center Bethel, Krankenhaus Mara, Bielefeld, Germany; ^5^Clinical Unit of Internal Medicine Small Animals, University of Veterinary Medicine, Vienna, Austria

**Keywords:** hippocampus, amygdala, tight junctions, limbic encephalitis, neuroinflammation

## Abstract

Human leucine-rich glioma-inactivated protein 1 encephalitis (LGI1) is an autoimmune limbic encephalitis in which serum and cerebrospinal fluid contain antibodies targeting LGI1, a protein of the voltage gated potassium channel (VGKC) complex. Recently, we showed that a feline model of limbic encephalitis with LGI1 antibodies, called feline complex partial seizures with orofacial involvement (FEPSO), is highly comparable to human LGI1 encephalitis. In human LGI1 encephalitis, neuropathological investigations are difficult because very little material is available. Taking advantage of this natural animal model to study pathological mechanisms will, therefore, contribute to a better understanding of its human counterpart. Here, we present a brain-wide histopathological analysis of FEPSO. We discovered that blood–brain barrier (BBB) leakage was present not only in all regions of the hippocampus but also in other limbic structures such as the subiculum, amygdale, and piriform lobe. However, in other regions, such as the cerebellum, no leakage was observed. In addition, this brain-region-specific immunoglobulin leakage was associated with the breakdown of endothelial tight junctions. Brain areas affected by BBB dysfunction also revealed immunoglobulin and complement deposition as well as neuronal cell death. These neuropathological findings were supported by magnetic resonance imaging showing signal and volume increase in the amygdala and the piriform lobe. Importantly, we could show that BBB disturbance in LGI1 encephalitis does not depend on T cell infiltrates, which were present brain-wide. This finding points toward another, so far unknown, mechanism of opening the BBB. The limbic predilection sites of immunoglobulin antibody leakage into the brain may explain why most patients with LGI1 antibodies have a limbic phenotype even though LGI1, the target protein, is ubiquitously distributed across the central nervous system.

## Introduction

In recent years, autoimmune epilepsies with antibodies against various antigens have been described. Among these are cases with antibodies targeting surface receptors such as the *N*-methyl-d-aspartate receptor (1), the α-amino-3-hydroxy-5-methyl-4-isoxazolepropionic acid receptor (2), the γ-aminobutyric acid-B receptor (GABA_B_R) (3), or the voltage-gated potassium channel (VGKC) complex (4). For the latter, it is now known that, in most cases, the antibodies are directed to leucine-rich glioma inactivated protein 1 (LGI1) (5, 6). Patients with LGI1 antibodies typically develop limbic encephalitis and suffer from amnesia, confusion, personality change or psychosis, seizures, and often hyponatremia (7, 8).

In human LGI1 encephalitis, magnetic resonance imaging (MRI) studies have shown increased volume and T2 signal of temporomesial structures with the hippocampus most prominently affected. Initial swelling of these structures is often followed by atrophy (8, 9). Whereas seizures, amnesia, and confusion likely result from autoantibody-mediated inflammation and lesions in the hippocampus (10), the neuropsychiatric symptoms, such as personality changes, psychosis or mood disorders, can be explained by the involvement of the amygdala, which is known for its key role in processing emotions and behavior (11–14).

The involvement of the hippocampus in human LGI1 encephalitis has been confirmed in histopathological studies. Parenchymal T cell infiltrates are present, but sparse. In addition, neurodegeneration caused by immunoglobulin G and complement deposition has been shown (15, 16). Two different pathogenic mechanisms may play a role in the action of LGI1 antibodies. First, the LGI1 antibodies seem to bind to LGI1, resulting in a functional change in LGI1, which can be reversed as steroids and plasma exchange swiftly ameliorate the disease symptoms (8, 17). Second, complement-mediated neuronal destruction occurring in the hippocampus may contribute to temporomesial atrophy and persisting cognitive deficits in nearly all patients, despite treatment options (9, 11, 15).

Recently, we described an acute seizure disorder with orofacial involvement in cats. This disorder was coined feline complex partial seizures with orofacial involvement (FEPSO) (18). Besides the distinct clinical features, these cats in MRI scans showed bilateral hippocampal T2 signal increase. Importantly, cell-based assays revealed that almost all tested sera of these cats contained anti-LGI1 antibodies (16, 18–20). Subsequent analysis of the hippocampus of these animals revealed the presence of inflammatory T cells, B cells, and plasma cells in the parenchyma. Moreover, neurodegeneration in these animals was caused by immunoglobulin and complement deposition (16). Overall, clinical, neuropathological and MRI analysis, therefore, indicate that FEPSO is the feline equivalent of human LGI1 encephalitis and might be used as a natural model for this disease.

In both human LGI1 encephalitis and in FEPSO, little is known about pathologic changes outside the hippocampus. Extrahippocampal pathology is expected since human LGI1 antibody-positive sera not only bind to the hippocampus but also to other regions, including the molecular layer of the cerebellum where LGI1 is expressed in high amounts (6, 21, 22). Nevertheless, in human patients, cerebellar features such as ataxia have been found much less frequently than limbic affection (23, 24). MRI studies in LGI1 encephalitis have shown T2 signal increase and sclerosis of the whole mesial temporal lobe (6, 8) but also in the basal ganglia, which are thought to contribute to faciobrachial dystonic seizures (25–27). Less frequently, white matter atrophy (28) and blurring, suggestive of mild de- or hypomyelination, was shown (29).

Taken together, although the limbic system displays the most frequent and most prominent signs and symptoms, recent publications point toward the involvement of other brain areas. The limited amount of human material, especially of brain structures other than the impaired hippocampus, poses a problem in the study of these extrahippocampal structures.

Since a relatively high number of FEPSO brains are available, we took the opportunity to perform a detailed neuropathological analysis of limbic and non-limbic structures. Our analysis shows that inflammatory infiltrates can be found brain-wide. A disturbance of the blood–brain barrier (BBB), occurring with prominent loss of tight junctions and resulting in leakage of immunoglobulin and complement, however, was more restricted. This leakage was found in brain areas such as the hippocampus but also in the subiculum, amygdale, and piriform lobe and, to a lesser degree, in the basal ganglia and hypothalamus. Moreover, in these areas, immunoglobulin and complement deposition to neurons was present and was associated with severe neurodegeneration. Importantly, this finding suggests that BBB disturbance and immunoglobulin leakage does not depend on T cell inflammation but that other, yet unknown, mechanisms are responsible.

## Materials and Methods

### Animals

In this study, 16 cats with FEPSO, characterized by facial seizures and hippocampal sclerosis or hippocampal necrosis (18), were included. Five of these cats were tested positive for serum antibodies against VGKC complex and showed positive reactivity to LGI1 in a cell-based binding assay as shown previously (16). The remaining 11 cats showed seizures typical of FEPSO but could not be tested for LGI1-reactive antibodies due to absence of sera. Throughout the manuscript, the definitive LGI1 antibody-positive FEPSO animals are indicated and separated from the FEPSO animals with unknown LGI1 antibody status. LGI1-positive animals are indicated in red and untested animals in black dots in all graphs. Of the 16 animals, 2 died spontaneously; all others were euthanized due to resistance to therapy or severe clinical course between 2 days and 34 months after onset of neurological signs. One cat (case 1) had papillary adenomas, whereas in the other cats, no tumors were found. Cats received antiepileptic therapy with phenobarbital, gabapentin, levetiracetam, potassium bromide, or a combination of those. Additionally, five cats were treated with prednisolone 1–2 mg/kg twice daily. For an epileptic control group, seven cats with epileptic seizures not fitting the classification of FEPSO (18) were used. Five suffered from temporal lobe epilepsy, four of which also had HS, one cat had an edema, and one a meningioma. Additionally, seven cats without neurological implication, which died as a result of other, non-neurological problems, were selected for the normal control group. For animal details, please refer to Table S1 in Supplementary Material.

### Ethics Approval

The project was discussed and approved by the institutional ethics committee (University of Veterinary Medicine, Vienna) in accordance with GSP guidelines and national legislation.

### Neuropathology and Immunohistochemistry

In all cats, a general necropsy was performed, and brains were fixed in 4% neutral-buffered formalin, embedded in paraffin, and coronal sectioned. Immunohistochemistry was performed as shown previously (15) at the level of the frontal cortex, nucleus accumbens, amygdala, hippocampus, and cerebellum. Luxol Fast Blue-Periodic Acid Schiff staining was performed to study changes (demyelination or hypomyelination) in white matter. Immunohistochemistry was performed with antibodies for T lymphocytes (anti-CD3), endothelial cells [transglutaminase 2 (TG2)], early constituents of the complement cascade (C1q), fully assembled complement system end complex (C9neo), feline immunoglobulin, and neurons [neuronal nuclei (NeuN)], and myelin-associated protein 2 (MAP-2). Antigen retrieval was done by heating the sections for 45 min in EDTA (0.05 M) in tris(hydroxymethyl)aminomethane (Tris) buffer (0.01 M, pH 8.5) or citrate buffer (0.01 M, pH 6) in a household food steamer device for all antibodies except for C9neo, and immunoglobulin, in which case antigen retrieval was performed by incubating the tissue for 15 min in proteinase (bacterial proteinase Type XXIV, #SLBQ7212V, Sigma Life Science) at 37°C. For more detailed information regarding antibodies, dilutions used, and antigen retrieval, please refer to Table S2 in Supplementary Material.

### Fluorescent Immunohistochemistry

We investigated BBB damage in more detail by double-labeling for the tight junction marker zona occludens 1 (ZO-1) and cat immunoglobulin in FEPSO animals in the hippocampus, the amygdala, basal ganglia, cortex, and cerebellum. Additionally, we investigated the hippocampus in normal and epileptic controls. Cats showing an average degree of BBB leakage were selected and investigated. To check for endothelial cell integrity of the blood vessels, we performed a triple staining for ZO-1, cat immunoglobulin, and von Willebrand Factor (vWF) as endothelial marker. Antigen retrieval was done with proteinase for 15 min at 37°C, and standard staining procedures were followed as described previously (15). For more detailed information regarding antibodies, dilutions used, and antigen retrieval, please refer to Table S2 in Supplementary Material.

### Terminal Deoxynucleotidyl Transferase dUTP Nick End Labeling

Qualitative assessment of chronic cell loss was conducted in stained coronal sections at the level of the frontal cortex, basal ganglia, amygdala, hippocampus, and cerebellum. For the detection of cells with DNA fragmentation, TUNEL staining was performed with the *In Situ* Cell Death Detection Kit^®^ (Roche, Basel, Switzerland) as described elsewhere (15) and developed with Fast Blue. To identify dying neurons, this step was followed by immunohistochemical staining for MAP-2 or NeuN, which was developed with 3-amino-9-ethylcarbazole as a substrate.

### Quantification of Cells

CD3^+^ cells were quantified by light microscopy using a morphometric grid in 1.25 mm^2^ (20 grids in 400× magnification) or 2.5 mm^2^ (40 grids in 400× magnification), depending on the number of brain slices containing the region of interest. C9neo^+^ cells were counted in 1.25 mm^2^ (20 grids in 400× magnification) for the cortex, cerebellum, and caudate nucleus. In the amygdala and the hippocampus, C9neo^+^ cells were counted in the whole area. For the determination of cell loss, the number of TUNEL^+^ cells among 100 cells was determined in the respective areas. For the determination of neuronal loss in the hippocampus, the number of NeuN^+^ cells was counted in 0.75 mm^2^ (3 grids in 200× magnification) of each hippocampal subfield in normal controls and FEPSO cats. The percentage of remaining NeuN^+^ cells in comparison with normal controls was calculated for each subfield. The statistical difference to 100% (equal to “no neuronal loss”) was calculated.

### Quantification of Immunoglobulin

For quantification of immunoglobulin in different brain areas as well as in the hippocampus between FEPSO and controls, all slides were incubated and developed for the final color reaction for the exact same time. Images were analyzed using ImageJ by digital optical densitometry, as shown previously (30).

### Magnetic Resonance Imaging

MR studies of two cats, acquired with a high-field MR unit (Magnetom Espree, 1.5T, Siemens Healthcare, Erlangen, Germany), were retrospectively evaluated. In each case, transverse T2-weighted fluid attenuation inversion recovery (FLAIR), sagittal 3D T2-weighted turbo spin echo (T2), transverse 2D and sagittal 3D pre- (T1) and post-contrast T1-weighted turbo spin echo images (T1C) were available. Slice thickness was 0.8–3 mm.

### Graphical Presentation of Inflammation, Neurodegeneration, and Complement Deposition

For a full overview on neuropathological changes, a brain-wide investigation for inflammation, neurodegeneration, and complement deposition was performed. Graphical representations of coronal cat brain slices containing the hippocampus, amygdala, cortex, basal ganglia, and cerebellum, were produced with CorelDRAW X4 based on images present on www.brainmaps.org (31). Infiltrates, neurodegeneration, and complement deposition in 16 cats with FEPSO were drawn into the cat brain images using Adobe Photoshop CS4.

### Statistical Analysis

For statistical analysis, GraphPad Prism 6 was used. First, we tested for differences between FEPSO animals positive for LGI1 antibodies and FEPSO animals with unknown LGI1 antibody status. To this end, a two-way ANOVA was used, but no differences were found. Therefore, datasets were pooled for further analysis, which was performed with Kruskal–Wallis tests with Dunn’s correction for multiple testing. Neuronal loss within the hippocampus was evaluated by the percentage of remaining neurons with regard to normal control hippocampal subareas (corresponding to 100% NeuN^+^ cells). To this end, a Wilcoxon-signed rank test was performed. All graphical data are represented as medians with the interquartile ranges. Animals tested positive for LGI1 antibodies and animals with unknown status are graphically separated (data points and error bars). Data of LGI1 antibody positive animals indicated in red and animals with unknown status indicated in black. Results were considered statistically significant at *p* ≤ 0.05.

## Results

### Inflammation

**Figure 1:**
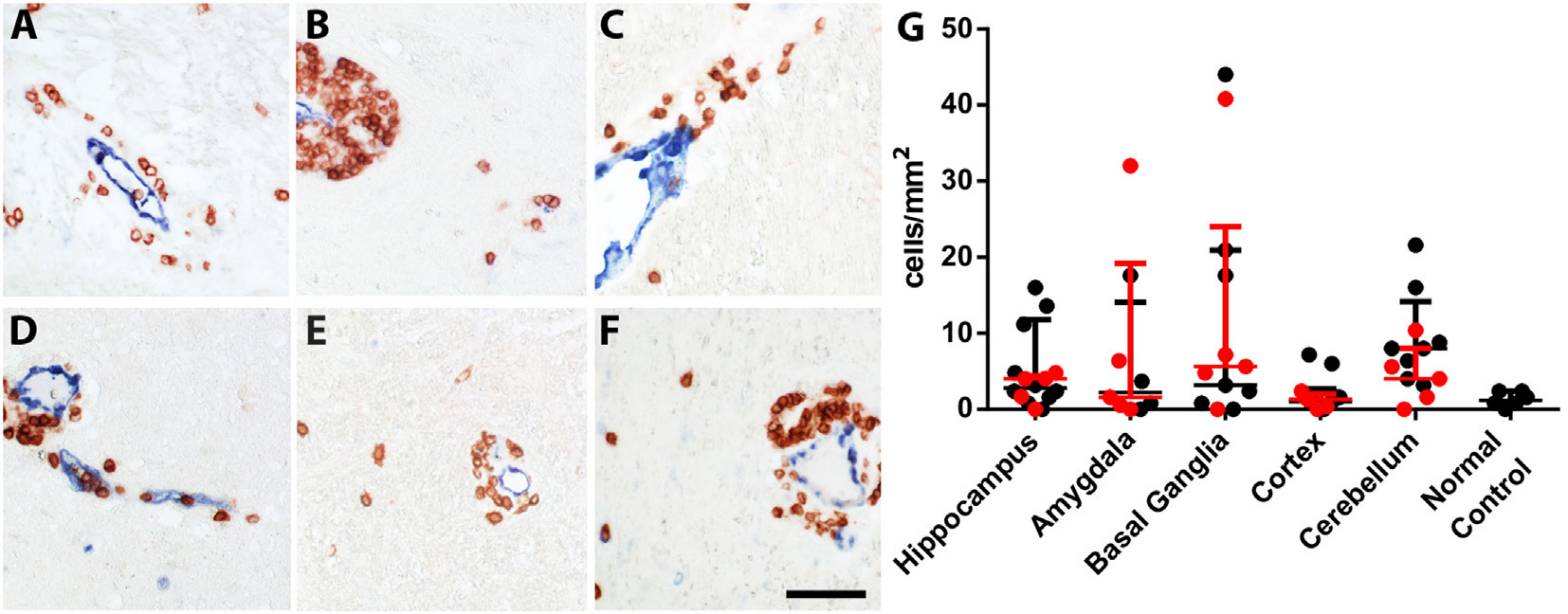
Inflammatory infiltrates in FEPSO can be found all over the brain in gray and white matter. Immunohistochemical staining for CD3^+^ and transglutaminase 2 showed that T cells are mainly located in perivascular cuffs with moderate parenchymal infiltrates in all inspected areas, namely the **(A)** hippocampus, **(B)** amygdala, **(C)** basal ganglia, **(D)** cortex, **(E)** cerebellum, and **(F)** white matter. **(G)** Quantification of parenchymal T cell infiltrates in the abovementioned areas did not reveal differences in T cell densities. Data shown as median with interquartile range, LGI1 antibody-positive animals are indicated in red and untested animals in black; (ns *p* > 0.05 Kruskal–Wallis test with Dunn’s *post hoc* correction, hippocampus *n* = 15, amygdala *n* = 9, basal ganglia *n* = 12, cortex *n* = 15, cerebellum *n* = 13, normal controls *n* = 6) Scale bar corresponds to 50 µm.

In the hippocampus of normal controls, parenchymal inflammatory T lymphocytes were very rare (1.2 cells/mm^2^). The hippocampus of epileptic controls showed higher numbers of T cells (2.4 cells/mm^2^). These T cell numbers were comparable to T cell numbers in hippocampi of FEPSO animals (3.2 cells/mm^2^). Statistical analysis of hippocampal T cell numbers between normal controls, epileptic controls, and FEPSO, possibly due to the high variance, however, revealed no significant difference. In brains of FEPSO animals, T cells were mostly found in perivascular cuffs and the meninges, with only moderate parenchymal infiltrating T cells. Parenchymal CD3^+^ T cells were found in all investigated regions of the brain, including the hippocampus, amygdala, basal ganglia, cortex, and cerebellum (Figures 1A–E and 6). T cell infiltrates, besides in gray matter, also were found in white matter tracts (Figure 1F). To determine if brain regions with high LGI1 abundance, such as the cerebellum or the hippocampus, showed increased levels of T cells, we quantified the number of CD3^+^ T cells in these different regions. However, no significant difference in cell number was found (Figure 1G).

### BBB Leakage

**Figure 2:**
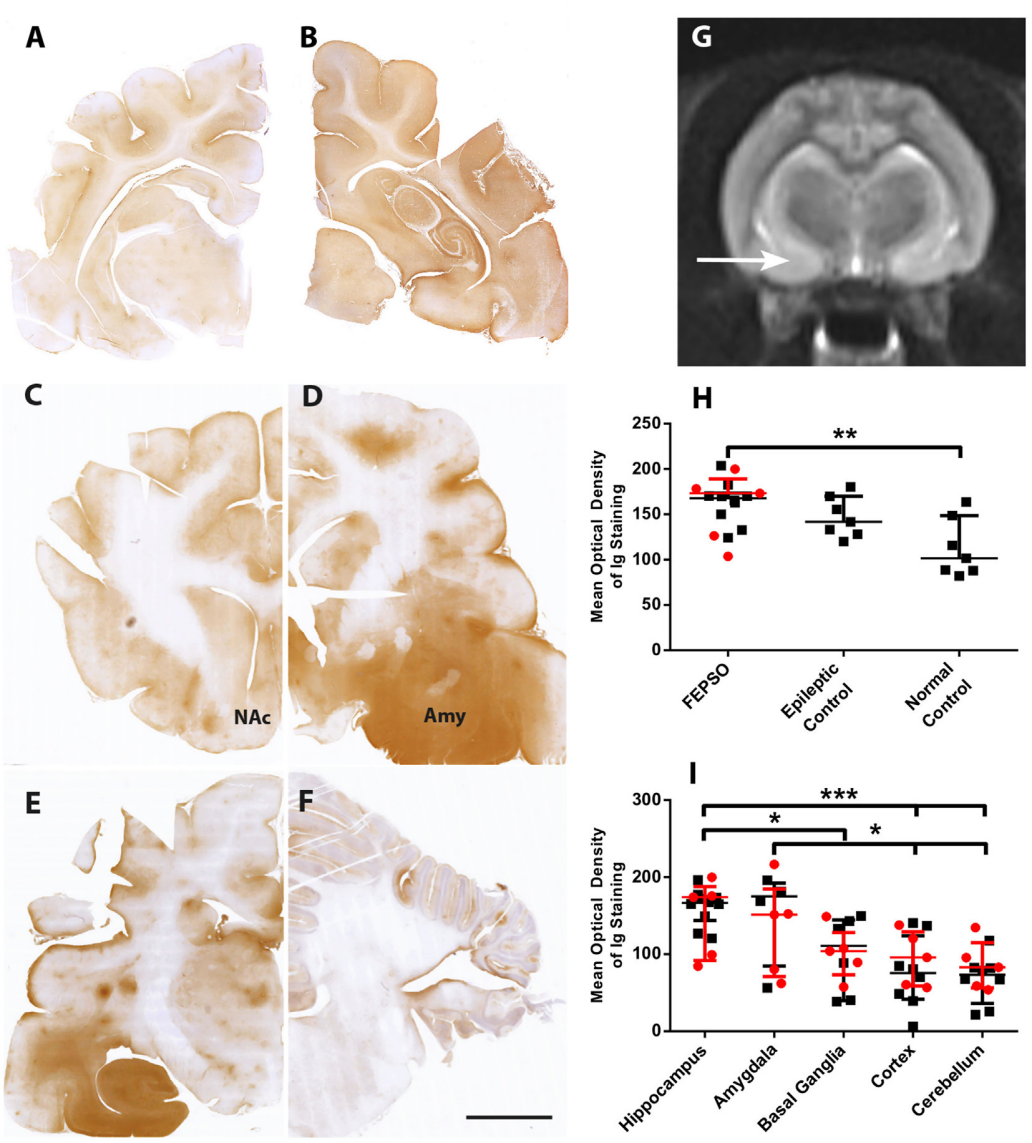
Selective blood–brain barrier leakage overlaps with MR signal changes in the amygdala. **(A–F)** Staining for immunoglobulin in representative images of coronal brain sections in **(A)** normal controls, **(B)** epileptic controls, and in FEPSO at the level of **(C)** frontal cortex and nucleus accumbens, **(D)** amygdala and piriform lobe, **(E)** hippocampus, and **(F)** cerebellum. Immunoglobulin leakage is seen prominently in the hippocampus but also in the amygdala and adjacent regions. Scale bar corresponds to 5 mm. **(G)** Transverse T2-weighted MR image at the level of the hippocampus and piriform lobe in a 1-year-old female neutered European shorthair cat, orientated along the long axis of the hippocampus on a sagittal slice. The MR signal is bilaterally abnormally increased, and the area of the amygdala (arrow) appears bilaterally enlarged. **(H)** Optical density quantification of Ig leakage in FEPSO, epileptic, and normal control hippocampus. The hippocampi of FEPSO have higher values than normal controls. Data shown as median with interquartile range, LGI1 antibody-positive animals, are indicated in red and untested animals in black (**p < 0.01, Kruskal–Wallis test with Dunn’s *post hoc* correction, FEPSO *n* = 15, epileptic controls *n* = 7, normal controls *n* = 7). **(I)** Significant elevation of immunoglobulin signal in hippocampus and amygdala in FEPSO cats compared to other brain areas of the same cats. LGI1 antibody-positive animals are indicated in red and untested animals in black. Data shown as median with interquartile range (*p < 0.05, ***p < 0.001; Kruskal–Wallis test with Dunn’s *post hoc* correction, hippocampus *n* = 15, amygdala *n* = 9, basal ganglia *n* = 12, cortex *n* = 15, cerebellum *n* = 13).

Immunoglobulin leakage was not found in the parenchyma of normal controls (Figure 2A). Epileptic controls, on the other hand, showed slightly increased levels of parenchymal immunoglobulin in the hippocampus and, to a lesser extent, in the cortex (Figure 2B). In FEPSO brains, leakage of immunoglobulin over the BBB was prominent in all limbic structures (Figures 2C–F). Most prominently affected were several nuclei of the amygdala, namely, the lateral and medial basal amygdala (N. basalis), the lateral (N. lateralis), medial (N. medialis) and central (N. centralis) amygdala, periamygdaloid area, and anterior cortical nucleus of the amygdala (N. corticalis) (Figure 2D), as well as, bilaterally, the hippocampus, adjacent subiculum, and entorhinal cortex (Figure 2E). These regions overlapped with signal and volume increase found in MR imaging (Figure 2G). In some animals, immunoglobulin leakage was also observed in the prepiriform cortex, putamen, claustrum, olfactory tubercle, nucleus accumbens, tenia tecta, septo-olfactory junction, hypothalamus, and anterior commissure (Figure 6). In the cerebellum, no significant immunoglobulin leakage was found (Figure 2F). Quantification of the mean optical density of immunoglobulin in the hippocampi confirmed the absence of immunoglobulin leakage of normal control cats. FEPSO cats showed a significant increase of immunoglobulin leakage compared to the baseline of normal controls (median of 70% increase). When compared with epileptic controls, no significant difference was found (Figure 2H). Within FEPSO animals, different brain areas showed large differences in immunoglobulin abundance. The amount of immunoglobulin in the hippocampus was significantly elevated compared to that in the basal ganglia, cortex, and cerebellum. Interestingly, we found no significant difference between the hippocampus and the amygdala, indicating comparable leakage in both areas. Moreover, the amount of immunoglobulin in the amygdala was significantly elevated compared with the cortex and the cerebellum (Figure 2I). We could not detect a significant difference in immunoglobulin signal intensities between hippocampal subareas.

### Magnetic Resonance Imaging

Because our study revealed pathological changes outside the hippocampus, especially in the amygdala, we analyzed MRIs from two FEPSO brains (#2 and #15) to support our findings. Bilateral T2-weighted signal and volume increase changes were found in the hippocampus as well as at the level of the amygdala and the piriform lobe (Figure 2G).

### Tight Junction Breakdown in Blood Vessels

**Figure 3:**
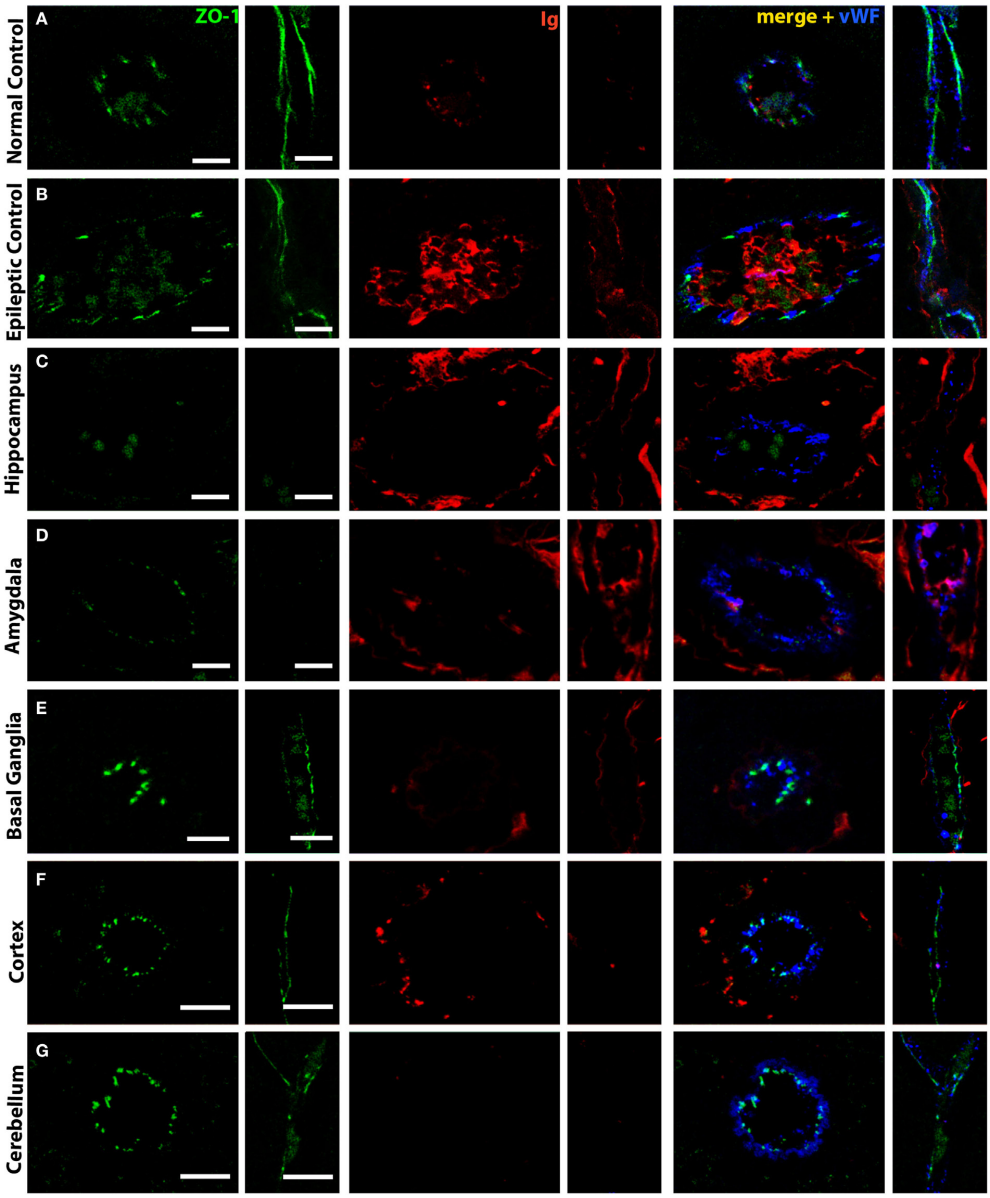
Selective tight junction breakdown leads to blood–brain barrier leakage. Staining of tight junctions [zona occludens 1 (ZO-1), green], immunoglobulin (red), and von Willebrand Factor (blue) in **(A)** normal controls, **(B)** epileptic controls, and **(C–G)** FEPSO cats positive for LGI1 antibodies. The tight junction protein ZO-1 is found between endothelial cells in a strong and (in longitudinal cuts) continuous staining in **(A)** normal controls in the hippocampus. Therefore, weak immunoglobulin staining is restricted to the luminal side of the blood vessels. **(B)** In hippocampi of epileptic controls, the ZO-1 staining is weaker, less continuous, and associated with some immunoglobulin reactivity in the parenchyma. **(C)** In FEPSO, blood vessels in the hippocampus have very weak and discontinuous ZO-1 signal, indicating tight junction breakdown. Here, immunoglobulin has clearly leaked into the parenchyma. **(D)** In the amygdala, the same pattern of tight junction breakdown was seen, indicated by a weak and discontinuous ZO-1 staining, and immunoglobulin leakage into the parenchyma. In all other investigated brain areas such as **(E)** basal ganglia, **(F)** cortex, and **(G)** cerebellum, in FEPSO cats, no ZO-1 breakdown was observed and immunoglobulin only was found on the luminal side of the vessels or perivascular space. Scale bars correspond to 10 µm **(A–D,F,G)** or 5 µm **(E)**.

Blood–brain barrier disruption might be associated with a loss of tight junctions. We, therefore, decided to investigate these structures in our animals. In control animals, the tight junction marker ZO-1 was strongly expressed around vessels. When viewed in longitudinal sections of capillaries, a continuous staining could be observed. Double labeling with Ig showed that, in such vessels, no leakage was observed and Ig was only seen on the luminal side of the vessel (Figure 3A). Under pathological conditions, in the hippocampus of epileptic controls, ZO-1 reactivity was weaker and discontinuous. Here, moderate immunoglobulin leakage could be observed in the surrounding brain parenchyma (Figure 3B). In the hippocampus of FEPSO animals, we observed a drastic decrease in ZO-1 reactivity. Here, ZO-1 immunoreactivity was very weak and visible in small patches instead of showing a continuous staining pattern. Furthermore, this loss of ZO-1 was associated with severe immunoglobulin leakage in the surrounding parenchyma (Figure 3C). The ZO-1 reactivity in the amygdala was comparable to what was seen in the hippocampus, with severe loss of ZO-1 intensity and loss of integrity. Additionally, also in the amygdala, strong immunoglobulin immunoreactivity was observed in the parenchyma, showing similar BBB breakdown and leakage as in the hippocampus (Figure 3D). This was different in basal ganglia (Figure 3E), cortex (Figure 3F), and cerebellum (Figure 3G) of FEPSO animals where ZO-1 reactivity was strong and in a regular continuous staining pattern. Immunoglobulin in these regions again was restricted to the lumen of the vessels, and Ig leakage in the parenchyma could not be found. To indicate the endothelial lining of the blood vessels, vWF was added in the merged images.

### Immunoglobulin and Complement Deposition

Normal control animals did not show any immunoglobulin deposition on neurons and epileptic controls showed immunoglobulin deposition in some cats. In FEPSO cats, brain areas with BBB leakage revealed strong membranous immunoglobulin deposition on severely damaged neurons. This was found in all limbic structures but was especially prominent in the hippocampus, (pre-) subiculum, entorhinal cortex, and amygdala. In some animals, immunoglobulin deposition was observed in the prepiriform cortex, putamen, claustrum, olfactory tubercle, nucleus accumbens, tenia tecta, septo-olfactory junction, hypothalamus, and anterior commissure. amygdala.

**Figure 4:**
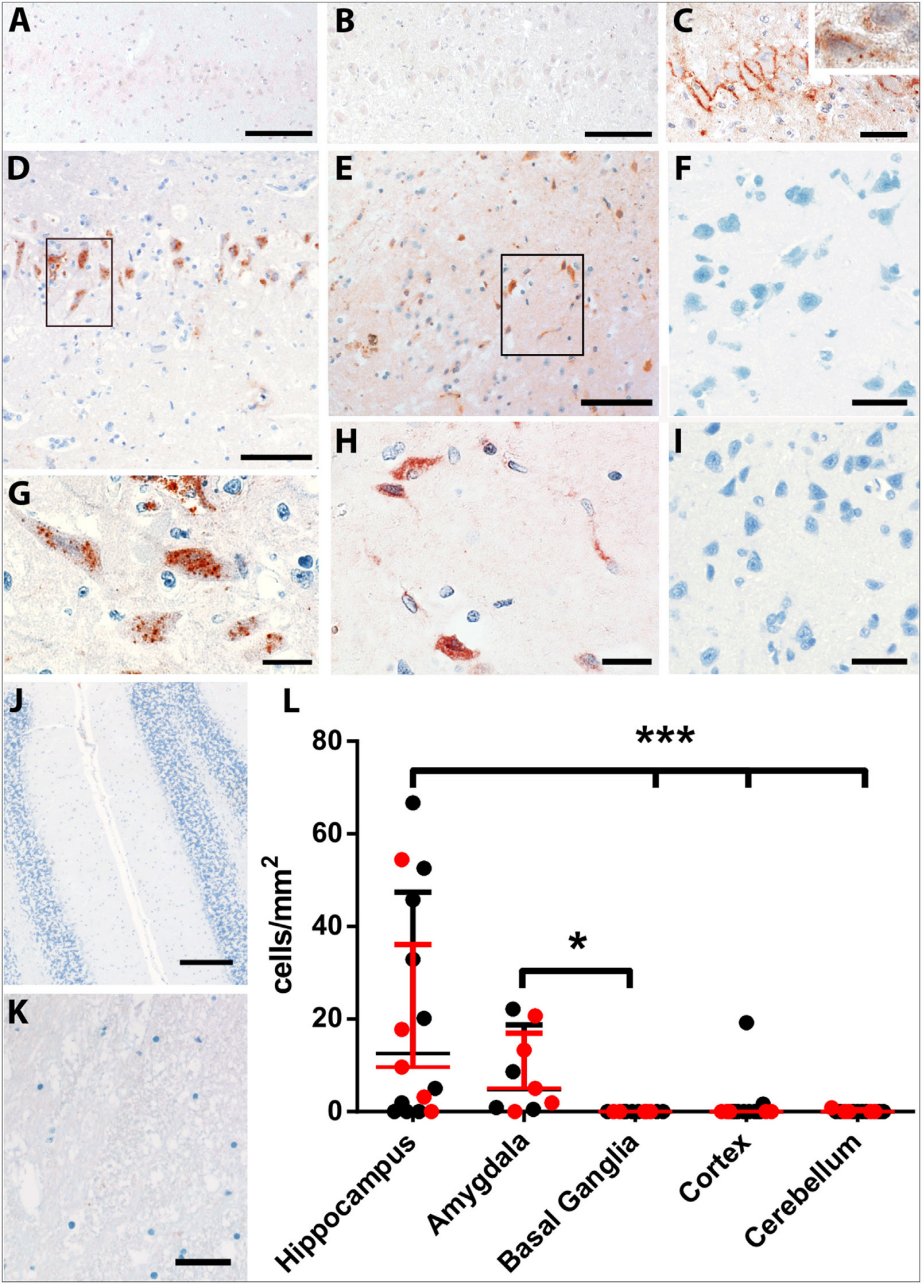
Complement deposition in LGI1 antibody-positive FEPSO animals. Staining for early constituent, C1q of the complement cascade shows the absence of C1q on neurons of **(A)** the hippocampus of a normal control and **(B)** the hippocampus of an epileptic control animal. **(C)** The hippocampus of a FEPSO cat shows C1q reactivity on the surface of hippocampal neurons with occasional granular staining inside the cells (inset). **(D–K)** The brain areas affected by BBB leakage are also affected by complement activation as shown by immunohistochemistry for C9neo. **(D)** In the hippocampus, complement-positive cells are visible, which show a [**(G)**, enlargement] granular staining pattern inside the cells. **(E)** The amygdala shows very similar staining with C9neo positive cells, which [**(H)**, enlargement] show the same granular staining inside the cells. Neither **(F)** the basal ganglia, **(I)** cortex, **(J)** cerebellum nor **(K)** white matter showed any complement positive cells or structures. **(L)** Quantification of C9neo positive cells revealed significantly higher numbers in the hippocampus as well as in the amygdala. Data shown as median with interquartile range, LGI1 antibody-positive animals are indicated in red, and untested animals in black (*p < 0.05, Kruskal–Wallis test with Dunn’s *post hoc* correction, hippocampus *n* = 15, amygdala *n* = 9, basal ganglia *n* = 12, cortex *n* = 15, cerebellum *n* = 13). Scale bars correspond to **(A,B,D,E)** 100 µm, **(C)** 50 µm, **(G,H)** 20 µm, **(F,I,K)** 25 µm, and **(J)** 200 µm.

In order to analyze early constituents of the complement cascade, we stained for complement factor C1q. In normal controls, C1q was occasionally found on the surface of cortical neurons. However, in none of these animals, C1q was found on neurons in the hippocampus (Figure 4A). A similar staining pattern was observed in epileptic controls (Figure 4B). In FEPSO animals, besides on the surface of some cortical neurons, C1q was mostly present on the surface of hippocampal neurons while single neurons in addition showed granular staining in the cytoplasm (Figure 4C).

Complement activation, indicated by the complement end complex marker C9neo, was not seen in normal controls. In epileptic controls, although immunoglobulin deposition was observed in some of these brains, C9neo reactivity was completely absent. In cats with FEPSO, regions with Ig deposition also showed complement C9neo immunoreactivity in a punctate appearance on the membrane as well as inside of neurons. This was most prominently seen in the hippocampus (Figures 4D,G) and in the amygdala (Figures 4E,H). Neither in the basal ganglia nor in the cerebellum were C9neo^+^ cells found (Figures 4F,J). Besides the entorhinal and prepiriform cortex, no complement deposition could be detected in the cortex (Figure 4I). Also, the white matter did not show any sign of complement activation (Figure 4K). In some animals, neurons with complement deposition were present in the dorsal hypothalamic area, tenia tecta, anterior prepiriform cortex, putamen, olfactory tubercle, and anterior commissure (Figure 6). Quantitative analysis of the number of C9neo^+^ neurons revealed that complement deposition was significantly elevated in the hippocampus and amygdala compared to other brain areas such as basal ganglia, cortex, and cerebellum (Figure 4L).

### Neurodegeneration and Neuronal Cell Loss

**Figure 5:**
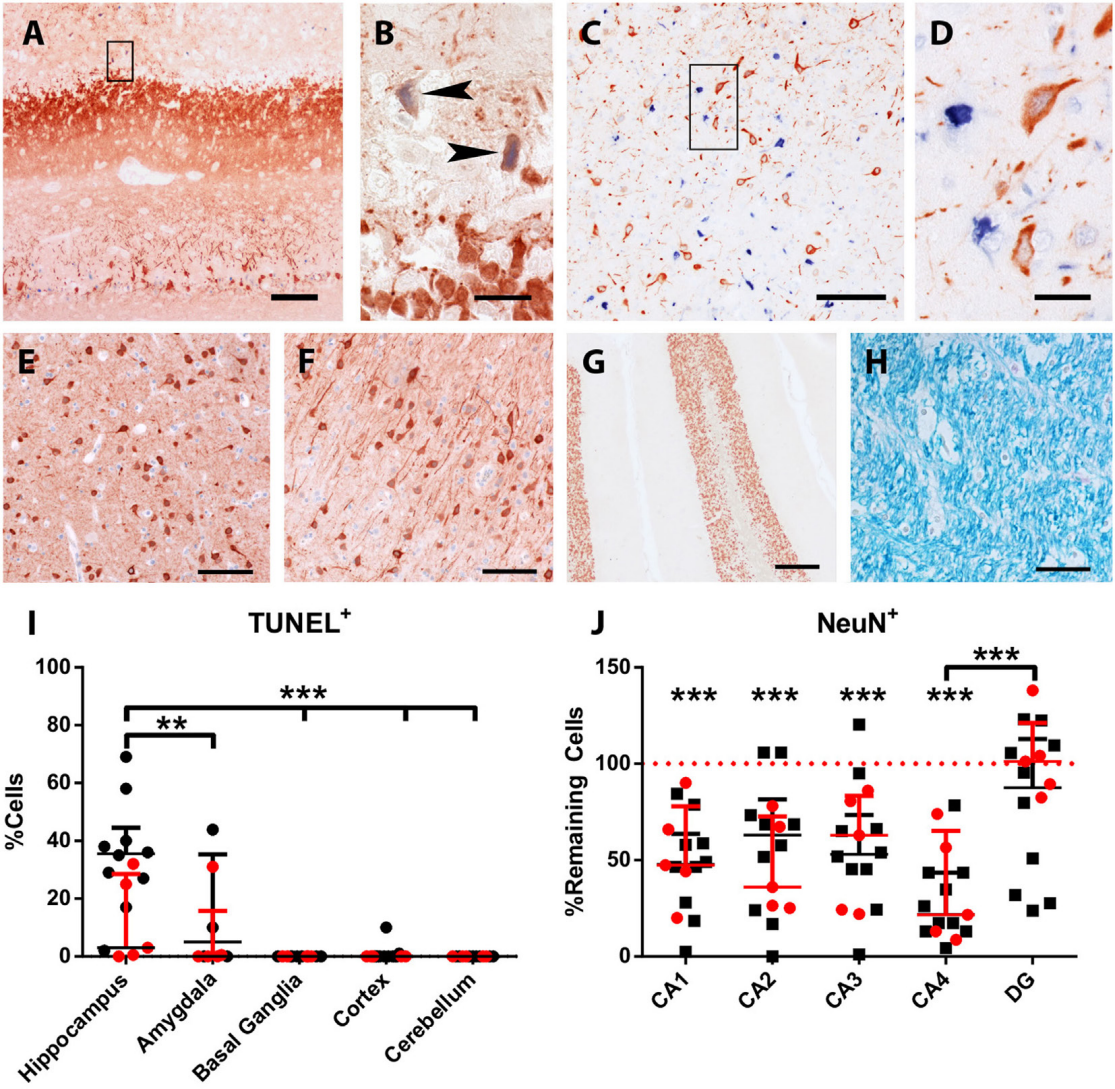
Acute neuronal degeneration and cell loss in LGI1 antibody-positive FEPSO animals. Staining for terminal deoxynucleotidyl transferase dUTP nick end labeling (TUNEL) (blue) and MAP2/neuronal nuclei (NeuN) (red). **(A)** TUNEL^+^ cells and severe loss of neurons and processes is visible in the hippocampus. **(B)** Shows the areas indicated by the rectangle in Figure 5A, revealing TUNEL^+^ neurons and loss of myelin-associated protein 2 reactivity (arrowheads). **(C)** In the amygdala, a high number of TUNEL^+^ nuclei are visible, while in **(D)**, enlargement of rectangle in Figure 5C and loss of MAP2-reactivity around TUNEL + nuclei is observed. Neither in the **(E)** basal ganglia, **(F)** cortex, **(G)** cerebellum nor **(H)** white matter TUNEL^+^ cells or de-hypomyelination was observed. **(I)** The percentage of TUNEL^+^ cells in different areas was quantified. This graph shows significant cell loss in the hippocampus of FEPSO animals. Data are shown as median with interquartile range, LGI1 antibody-positive animals are indicated in red, and untested animals in black (***p < 0.001, Kruskal–Wallis test with Dunn’s *post hoc* correction, hippocampus *n* = 15, amygdala *n* = 9, basal ganglia *n* = 12, cortex *n* = 15, cerebellum *n* = 13). **(J)** Hippocampal cornu ammonis (CA) subfields revealed severe loss of NeuN-positive cells compared with normal controls (equals 100%) in CA1-4, but not in DG. CA4 subfields exhibits reveal the most severe cell losses, which was significantly different from the loss in the DG. Data shown as median with interquartile range (***p < 0.001, Wilcoxon signed-rank test with hypothetical value: 100, *n* = 14, **p < 0.01, Kruskal–Wallis test with Dunn’s *post hoc* correction for evaluation of inter-hippocampal differences, *n* = 14). Scale bar corresponds to **(A)** 200 µm, **(B,D)** 20 µm, **(C)** 100 µm, **(E,F,H)** 50 µm, and **(G)** 200 µm.

**Figure 6:**
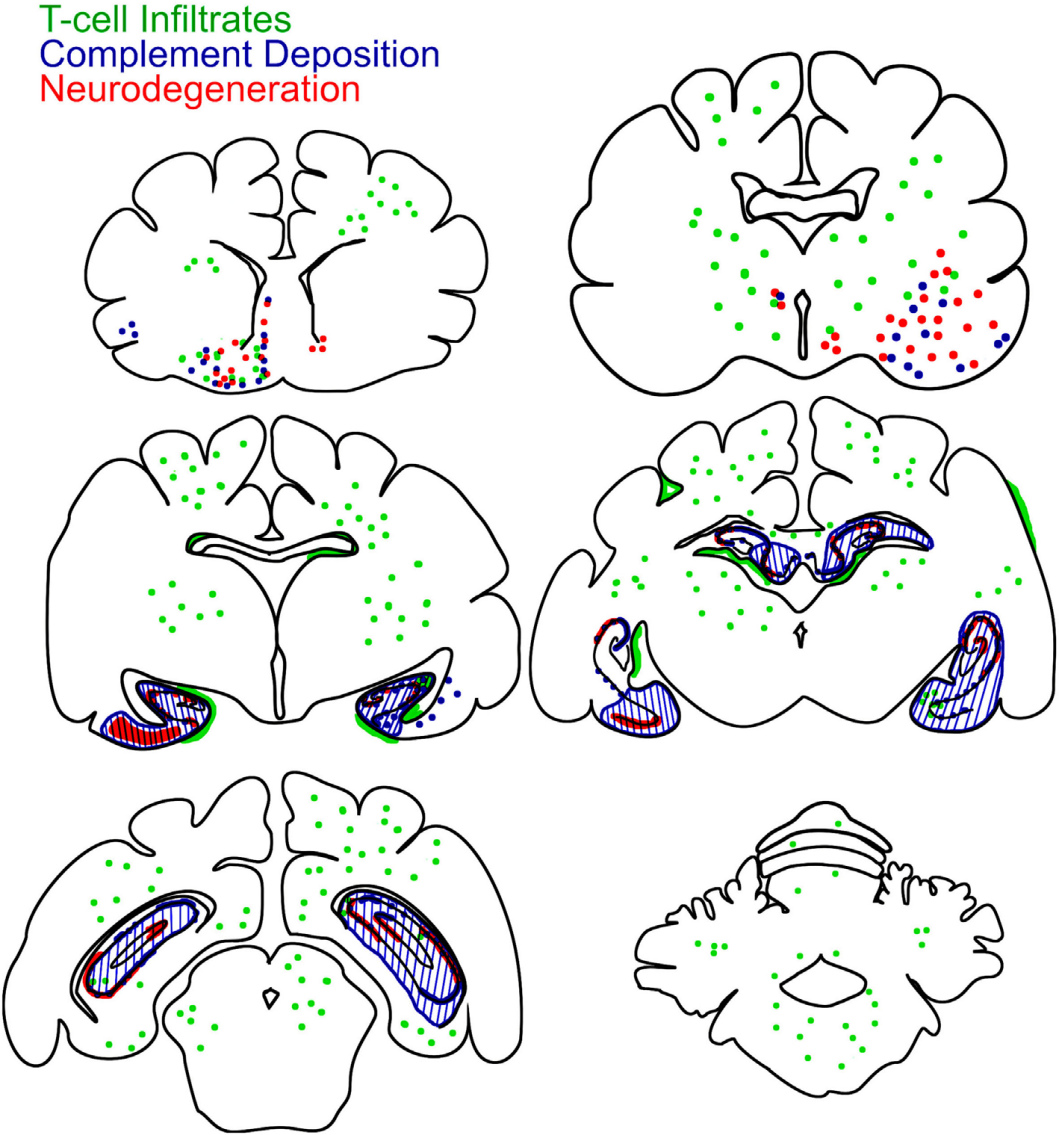
Brain-wide mapping of pathological changes in FEPSO. Cumulative graphical presentation of T cell infiltrates (green), neurodegeneration (red), and complement deposition (blue) in the brains of 16 cats suffering from FEPSO. This presentation shows that inflammatory lesions are brain-wide. Neurodegeneration and complement deposition show strong overlap and is most prominent in the hippocampus, amygdale, and adjacent areas.

None of the normal control animals showed neuronal loss. Epileptic control animals, however, showed variable neuronal loss in the hippocampus and subiculum. In FEPSO animals, regions showing neurodegeneration largely correlated with immunoglobulin and C9neo deposition and were found in both hemispheres in the limbic structures (Figure 6). The hippocampus and the subiculum showed high numbers of TUNEL^+^ cells, indicating a high degree of cell death (Figures 5A,B). Additionally, we found TUNEL^+^ cells in the amygdala (Figures 5C,D). The affected areas comprised the lateral and medial basal amygdala (N. basalis), the lateral (N. lateralis), medial (N. medialis) and central (N. centralis) amygdala, periamygdaloid area, and anterior cortical nucleus of the amygdala (N. corticalis). In single animals, we also found neuronal loss and some TUNEL^+^ cells in the tenia tecta, septo-olfactory junction, entorhinal cortex, anterior commissure, nucleus accumbens, claustrum, or the posterior prepiriform area (Figure 6). In none of the FEPSO animals was neurodegeneration observed in the cortex (except the entorhinal cortex, see above) or cerebellum (Figures 5E–G). Moreover, the white matter did not show any sign of hypo- or demyelination (Figure 5H). Quantification of the number of TUNEL^+^ cells showed a significant increase of cell death in the hippocampus compared with the amygdala, basal ganglia, cortex, and cerebellum (Figure 5I).

Additionally, we questioned whether hippocampal subfields were equally affected. Quantification of neurons in these subfields in normal controls and FEPSO showed that CA4 was the most severely affected (median cell loss of 78.3%). In CA1, 52.2% were lost, and in CA2 and CA3, 37.5 and 46.7% were lost, respectively. The dentate gyrus, with a median of 98.2% remaining neurons, did not show significant neuronal loss, although three FEPSO animals also showed severe neurodegeneration in the dentate gyrus with less than 50% of neurons remaining. Finally, CA4 showed significant neuronal loss compared with the dentate gyrus (Figure 5J). This neuronal loss in FEPSO corresponds to human hippocampus sclerosis type 1 according to the classification of the international league against epilepsy (32). One recent MRI study on human LGI1 encephalitis found volume loss in CA3 only (33), whereas another one found all areas except CA1 to be affected (9). These intrahippocampal differences, however, cannot be explained by differential expression of LGI1, as, at least in mice, it is most prominently expressed in CA3 and the dentate gyrus (22), and these two subareas in FEPSO strongly differ in the amount of neurodegeneration.

## Discussion

LGI1 encephalitis is a form of autoimmune epilepsy, an expanding group of disorders with antibodies against various neural antigens and epileptic seizures as one of the broad range of neurological signs. The similarities between human LGI1 encephalitis and FEPSO have only recently been discovered (16, 20). These similarities comprise not only the presence of antibodies against LGI1 and the pathological changes seen in the hippocampus but also clinical symptoms such as radiological changes and facial seizures (15, 16).

The findings that human LGI1-positive sera show strong staining of the cerebellum and that human patients can (very rarely) develop cerebellar symptoms (24, 34) and the MRI studies that show involvement of the amygdala (35, 36) and basal ganglia (25–27) piqued our interest to investigate pathological changes in brain structures other than the hippocampus. Here, we show that inflammatory infiltrates are not restricted to the hippocampus but are also found in most other parts of the brain. Since inflammatory lesions do not overlap with areas affected by neurodegeneration, it can be assumed that T cells do not directly contribute to cell death. Interestingly, in human VGKC-limbic encephalitis, T cell infiltrates have also been found in the amygdala and the uncus in similar numbers as in FEPSO (15). The number of these infiltrating T cells is much lower than those found in T cell-mediated neurodegeneration, such as in paraneoplastic encephalitis in humans (15). In line with our results, a human MRI study found brain-wide metabolic changes, in gray as well as white matter, pointing toward brain-wide neuroinflammation (28). Here, we also found prominent T cell infiltrates in the white matter. This might explain the recently reported supratentorial white-matter blurring in MRI scans of human LGI1 encephalitis brains (29), since myelin breakdown and inflammatory infiltrates leading to localized edema can be difficult to distinguish in MRI scans (37).

Interestingly, in FEPSO, BBB disturbance was not purely restricted to the hippocampus but was also revealed in presubiculum, subiculum, amygdala, piriform lobe and, more rarely, in areas such as basal ganglia and hypothalamus. The larger limbic areas were also found to be affected in MR T2-weighted FLAIR scans showing signal as well as volume increase. In human LGI1 encephalitis, hippocampal and amygdaloidal swelling, followed by atrophy of the affected areas, have been observed in MRI studies (35, 36, 38). Subsequently, considering the similarities in histopathology between human and feline hippocampi, and considering similar MRI changes in the human and feline hippocampus as well as amygdala, it can be assumed that in human LGI1 encephalitis, the amygdala might be similarly affected by BBB leakage and neurodegeneration as shown here in cats. Among patients with anti-LGI1 encephalitis, hypothalamic dysfunction in form of hyponatremia due to the syndrome of inappropriate antidiuretic hormone secretion [~60% of patients (5, 6)] and basal ganglia involvement in form of faciobrachial dystonic seizures [~50% of patients (8)] are readily known. Cerebellar affects have only rarely been reported in human LGI1 encephalitis cases (5, 23, 24). In our cats, hyponatremia and neurodegeneration of the hypothalamus as well as basal ganglia was seen occasionally. On the other hand, no cerebellar symptoms were observed, and we found neither BBB leakage and complement activation nor neurodegeneration in the cerebellum.

An important finding in this study of FEPSO is that BBB leakage was more restricted to specific areas associated with the limbic system, whereas T cell infiltrates were present brain wide. A clear finding in animal models of multiple sclerosis and neuromyelitis optica is that activated T cells can open the BBB and thereby enable antibodies to enter the CNS (39–44). Here, in FEPSO, however, T cell infiltrates and BBB leakage do not seem to overlap, since brain areas with high LGI1 abundance, such as the cerebellum (22), are spared of complement activation and neurodegeneration, despite a comparable number of infiltrating T cells. Therefore, this finding point toward another mechanism or an additional trigger that is required to induce local disturbance of the BBB. It has been shown repeatedly that cytokines, peripheral, or in the CNS, have a drastic effect on BBB permeability (45–49). Intrastriatal injection of interleukin-1β has been shown to profoundly alter BBB permeability, leading to complement activation in Lewis rats (46). Interferon-γ and tumor necrosis factor-α have been shown to decrease tight junction proteins, such as occludin (47, 50). Moreover, oxidative stress and cytokines have been reported to change phosphorylation patterns of various tight junction proteins, among them occludin, e-cadherin, and ZO-1, thereby dissociating the tight junction complexes (51, 52). However, to explain the selective breach of the BBB in FEPSO, other factors can also come into play. These are, for example, differences in permeability of the BBB, which have been reported in mice (53, 54). Moreover, differential expression of receptors on the microvasculature in the brain can lead to different alterations in BBB permeability (55). Furthermore, external factors can also be involved in the opening of the BBB. High adrenaline levels caused by trauma or extreme exercise, cocaine, and nicotine have an effect on BBB permeability (55). Animal studies with NR2-antibodies showed that additional factors, such as lipopolysaccharides and epinephrine, can open the BBB in distinct locations and, therefore, the same antibodies can cause different symptoms (56, 57). Finally, here, we showed increased immunoglobulin leakage in the hippocampus of cats with LGI1 antibodies when compared with normal control cats. However, compared with epileptic controls, no difference was found. This is not surprising as it has been shown repeatedly that BBB dysfunction can result in epilepsy and seizures, which in turn can lead to disturbances in the BBB, resulting in a vicious circle (58–62).

To summarize, in this naturally occurring animal model, we were able to show that BBB disturbance and neurodegeneration in FEPSO is restricted to the hippocampus and other limbic structures, but is not found in other areas such as the cerebellum despite the presence of T cell infiltrates. Our findings broaden the perspective on LGI1 encephalitis by not only looking at the target organ (the brain parenchyma) but also on the question of how the presumably pathogenic antibodies can enter it. This not only deepens the knowledge and understanding of LGI1 encephalitis but also leads to new questions regarding regional selectivity of dysfunctional BBB. These findings are not only of use in veterinary medicine but also are highly relevant to human disease. A better understanding of underlying disease mechanisms will help to ameliorate future treatments and hopefully minimize remaining cognitive deficits of patients.

## Ethics Statement

The project was discussed and approved by the institutional ethics committee (University of Veterinary Medicine, Vienna) in accordance with GSP guidelines and national legislation. In addition, all animal owners have given their consent.

## Author Contributions

JB and AP designed and supervised the project. AT and JB prepared the manuscript. AT performed the statistical analysis and, together with JB, the data interpretation. AT, MF, and LQ-G performed immunohistochemical staining, cell quantification, and imaging. AK performed the necropsies. SK provided MRI scans and interpretation of those. CB contributed to the study design. All authors reviewed the manuscript.

## Conflict of Interest Statement

CB gave scientific advice to Eisai (Frankfurt, Germany) and UCB (Monheim, Germany), undertook industry-funded travel with support of Eisai (Frankfurt, Germany), UCB (Monheim, Germany), Desitin (Hamburg, Germany), and Grifols (Frankfurt, Germany), obtained honoraria for speaking engagements from Eisai (Frankfurt, Germany), UCB (Monheim, Germany), Desitin (Hamburg, Germany), Diamed (Köln, Germany), Fresenius Medical Care (Bad Homburg, Germany), Biogen (Ismaning, Germany), and Euroimmun (Lübeck, Germany). He received research support from Diamed (Köln, Germany) and Fresenius Medical Care (Bad Homburg, Germany). He is a consultant to the Laboratory Krone, Bad Salzuflen, Germany, regarding neural antibodies and therapeutic drug monitoring for antiepileptic drugs. The other authors declare no competing financial interests.

## Abbreviations

AMPAR, α-amino-3-hydroxy-5-methyl-4-isoxazolepoionic acid receptor; BBB, blood–brain barrier; FEPSO, feline complex partial seizures with orofacial involvement; FLAIR, Fluid attenuation inversion recovery; vWF, von Willebrand factor; GABA_B_R, gamma aminobutyric acid B receptor; HN, hippocampal necrosis; HS, hippocampal sclerosis; LGI1, leucine-rich glioma-inactivated protein 1; MAP-2, myelin-associated protein 2; NeuN, neuronal nuclei; NMDAR, *N*-methyl-d-aspartate receptor; TG2, transglutaminase 2; TUNEL, terminal deoxynucleotidyl transferase dUTP nick end labeling; VGKC, voltage-gated potassium channel complex; ZO-1, zona occludens 1.
