# Supplementary material for: Selective Limbic Blood–Brain Barrier Breakdown in a Feline Model of Limbic Encephalitis with LGI1 Antibodies
Source: Front Immunol. 2017 Oct 18;8:1364. doi: 10.3389/fimmu.2017.01364 (PMC5651237; doi:10.3389/fimmu.2017.01364)
Supplement: Supplementary file 1 [file Table_1.DOCX]

Supplementary Material

**Selective Limbic Blood-Brain Barrier Breakdown in a Feline Model of Limbic Encephalitis with LGI1 Antibodies**

**Anna R. Tröscher^1^, Andrea Klang^2^, Maria French^1^, Lucía Quemada-Garrido^1^, Sibylle Kneissl^3^, Christian G. Bien^4^, Ákos Pákozdy^5,#^, Jan Bauer^1,#,*^**

Corresponding author:

Dr. Jan Bauer

Email: [jan.bauer@meduniwien.ac.at](mailto:jan.bauer@meduniwien.ac.at)

**Supplementary Table 1**

Supplementary Table 1: Animal Details

| Animal No | Age (y) | Sex (M/F) | Breed | Diagnosis | Therapy | Disease Duration | MRI | VGKC/  LGI1 Titer (pmol/L) |
| --- | --- | --- | --- | --- | --- | --- | --- | --- |
| FEPSO |  |  |  |  |  |  |  |  |
| 1 | 11.1 | F | ESH | FEPSO+Ad | Anti-E+crt | 5 d | ND | 123/+ |
| 2 | 2.5 | F | ESH | FEPSO | Anti-E+crt | 30 d | HS + A | 443/+ |
| 3 | 7 | M | ESH | FEPSO | Anti-E+crt | 89 d | NP | 791/+ |
| 4 | ad | F | ESH | FEPSO | No tx | 2 d | NP | 24/NP |
| 5 | 2 | F | ESH | FEPSO | Anti-E | 8 d | NP | NP |
| 6 | 2.3 | F | ESH | FEPSO | Anti-E | 10 d | NP | NP |
| 7 | 4.3 | M | Siam | FEPSO | Anti-E | 3 d | NP | NP |
| 8 | 4.9 | M | ESH | FEPSO | Anti-E | 49 d | NP | NP |
| 9 | 14 | F | ESH | FEPSO | Anti-E | 91 d | NP | NP |
| 10 | 3 | M | ESH | FEPSO | Anti-E | 2 y 10 m | NP | NP |
| 11 | 7.5 | M | ESH | FEPSO | No tx | 4 m | NP | NP |
| 12 | 11 | F | ESH | FEPSO | No tx | unknown | NP | NP |
| 13 | 1 | F | ESH | FEPSO | Anti-E | 6 d | NP | NP |
| 14 | 7.6 | M | ESH | FEPSO | No tx | 3 d | NP | NP |
| 15 | 12.8 | F | ESH | FEPSO | Anti-E+crt | 78 d | HS + A | 686/+ |
| 45 | 6.1 | F | ESH | FEPSO | Anti-E+crt | 56 d | NP | 340/+ |
| Epileptic Co |  |  |  |  |  |  |  |  |
| 16 | 3 | F | ESH | TLE and HS | ND | 7 d | NP | NP |
| 17 | 8 | F | Car | TLE and HS | ND | 3 d | NP | NP |
| 18 | 1.5 | M | ESH | TLE and HS | ND | 23 d | NP | NP |
| 26 | 1.3 | M | ELH | Edema | ND | 1 h | NP | NP |
| 27 | 13 | M | Persian | TLE and HS | ND | 16 d | NP | NP |
| 28 | 1.2 | M | HK | TLE | ND | 1 y | NP | NP |
| 29 | 18 | F | EKH | Meningioma | ND | 20 d | NP | NP |
| Normal Co |  |  |  |  |  |  |  |  |
| 24 | ad | F | Ben | CO | No tx | 0 d | NP | NP |
| 25 | 0.6 | M | BSH | CO | No tx | 0 d | NP | NP |
| 30 | 0.7 | F | EKH | CO | No tx | 0 d | NP | NP |
| 31 | 0.6 | F | EKH | CO | No tx | 0 d | NP | NP |
| 32 | 1.5 | F | EKH | CO | No tx | 0 d | NP | NP |
| 33 | 0.6 | M | EKH | CO | No tx | 0 d | NP | NP |
| 34 | 0.6 | M | BSH | CO | No tx | 0 d | NP | NP |

Abbreviations: F: female; M: male;

Co: Control

Breed: Ben: Bengal cat, BSH: British short-hair cat, Car: Carthusian cat, ELH: European long-hair cat, ESH: European short-hair cat, Siam: Siamese cat,

Age: ad: adult

Diagnosis: FEPSO: feline complex partial seizures with orofacial involvement; FEPSO+Ad: feline complex partial seizures with orofacial involvement and adenoma; TLE, HS: Temporal lobe epilepsy with hippocampus sclerosis

Therapy: no tx: no treatment, anti-E: anti-epileptic treatment, crt: corticosteroid, ND: no data

Disease duration: d: days, m: months, y: years

MRI: Magnetic resonance imaging, HS+A: Hippocampal sclerosis with amygdala swelling, NP: not performed

VGKC: serum anti-VGKC antibody concentration as determined by radioimmunoprecipitation.

LGI1: serum LGI1 antibody status based on indirect immunofluorescence on transfected HEK cells; +: positive, -: negative
