# Supplementary material for: Selective Limbic Blood–Brain Barrier Breakdown in a Feline Model of Limbic Encephalitis with LGI1 Antibodies
Source: Front Immunol. 2017 Oct 18;8:1364. doi: 10.3389/fimmu.2017.01364 (PMC5651237; doi:10.3389/fimmu.2017.01364)
Supplement: Supplementary file 2 [file Table_2.DOCX]

Supplementary Material

**Selective Limbic Blood-Brain Barrier Breakdown in a Feline Model of Limbic Encephalitis with LGI1 Antibodies**

**Anna R. Tröscher^1^, Andrea Klang^2^, Maria French^1^, Lucía Quemada-Garrido^1^, Sibylle Kneissl^3^, Christian G. Bien^4^, Ákos Pákozdy^5,#^, Jan Bauer^1,#,*^**

Corresponding author:

Dr. Jan Bauer

Email: [jan.bauer@meduniwien.ac.at](mailto:jan.bauer@meduniwien.ac.at)

**Supplementary Table 2**

Supplementary Table 2: Antibodies used for immunohistochemistry

| Antigen | | | Host Species | Final Dilution and Antigen Retrieval | | Source | | |
| --- | --- | --- | --- | --- | --- | --- | --- | --- |
| C9neo | | | Rat | 1:2000  Proteinase (15 min)  Incubation o/n at 4°C | | Kind gift from S. Piddlesden, University of Cardiff, UK | | |
| Feline Immunoglobulin  Cy3 directly conjugated | | | Goat | Light microscopy:  1:500  Fluorescence: 1:250  Proteinase (15 min)  Incubation o/n at 4°C | | Jackson Immunoresearch,  102-165-003 | | |
| CD3 | | | Rabbit | 1:1000  Citrate (45 min)  Incubation o/n at 4°C | | DakoCytomation,  A0452 | | |
| NeuN | | | Mouse | 1:500  Citrate (30 min)  Incubation o/n at 4°C | | Chemicon/Millipore  MAB377 | | |
| Map2 | | | Mouse | 1:100  EDTA 8,5 (30 min)  Incubation o/n at 4°C | | Sigma  M4403 | | |
| ZO-1 | | | Rabbit | 1:50  Proteinase (15 min)  Incubation o/n at 4°C | | Invitrogen  61-7300 | | |
| TG2 | | | Mouse | 1:1500  EDTA 8,5 (45 min)  Incubation o/n at 4°C | | NeoMarkers  Ab3 | | |
| vWF | | | Rabbit | 1:300  Proteinase (15 min)  Incubation o/n at 4°C | | Dako  #A0082 | | |
| C1q α-Chain | | | Goat | 1:50  Citrate (45 min)  Incubation o/n 4°C | | Santa Curz  #sc-27661 | | |
| Biotin-α-rat | | Donkey | | 1:1500  Incubation 1h at RT | | Jackson ImmunoResearch  #712-065-153 | | |
| Biotin-α-goat | | Donkey | | 1:500  Incubation 1h at RT | | Jackson ImmunoResearch  #705-065-147 | | |
| Biotin-α-rabbit | | Donkey | | 1:2000  Incubation 1h at RT | | Jackson ImmunoResearch  #711-065-152 | | |
| Biotin-α-mouse | | Donkey | | 1:1500  Incubation 1h at RT | | Jackson ImmunoResearch  #705-065-150 | | |
| Alkaline Phosphatase - α-mouse | Donkey | | | 1:100  Incubation 1h at RT | | Jackson Immuno Research  #715-055-151 | | |
| Cy5- α-Rabbit | Donkey | | | 1:200  Incubation 1h at RT | | Jackson Immuno Research  #711-175-152 | | |
|  |  | | |  | |  | | |
| Streptavidin-Cy2 | |  | | 1:100  Incubation 1h at RT | | Jackson ImmunoResearch  #016-220-084 | | |
| CD31* | | Mouse | | 1:25  EDTA 8,5 (45 min) and Proteinase (15 min)  Incubation o/n 4°C | Dako JC70A | | |  |
| CD31* | | Rabbit | | 1:200  EDTA 8,5 (45 min) and Proteinase (15 min)  Incubation o/n 4°C | Abcam #28364 | | |  |
| CD34* | | Mouse | | 1:25  EDTA 8,5 (45 min) and Proteinase  (15 min)  Incubation o/n 4°C | Novocastra NCL-END | | |  |
|  |  |  |  |  |  | | |  |
| Isolectin+POX* | | Bandeiraea simplicifolia | | 1:50  Citrate (45 min) and Proteinase (15 min)  Incubation o/n 4°C | Sigma –Aldrich  L2895 | | |  |
| NG2* | | Rabbit | | 1:250  EDTA 8,5 (45 min) and Proteinase (15 min)  Incubation o/n 4°C | Chemicon-Millipore #AB5320 | | |  |
| PECAM * | | Mouse | | 1:200  EDTA 8,5 (45 min) and Proteinase (15 min)  Incubation o/n 4°C | Neomarkers Ab-1 | | |  |
| Laminin* | | Mouse | | 1:20  Without antigen retrieval and Proteinase (15 min)  Incubation o/n at 4°C | Dako M0638 | | |  |
|  |  |  |  |  |  | | | |
|  |  | | |  |  | |  | |
| C3* | Rabbit | | | 1:150 and 1:1000  Citrate (45 min)  Incubation o/n at 4°C | Dako A0063 | |  | |
| C5b* | Rat | | | 1:1000  EDTA 8,5 (45 min)  Incubation o/n at 4°C | Hycult clone 21 | |  | |

# *Antibodies did not show specific staining on cat tissue

# Abbreviations: EDTA: Ethylenediaminetetraacetic acid; RT: room temperature
